# From Synapsia to Ideas: Nitric Oxide and Sensory Roots of Knowledge

**DOI:** 10.33549/physiolres.74.S169

**Published:** 2025-12-01

**Authors:** Fedor Jagla, Olga Pecháňová

**Affiliations:** Centre of Experimental Medicine, Institute of Normal and Pathological Physiology, Slovak Academy of Sciences, Bratislava, Slovakia

*“Nihil est in mind quot nos prius fuerit in sensu.”*

*(John Locke: Essay About Human Reason, 1690)*

The famous Czech writer Milan Kundera in his essay The Abused Heritage of Cervantes wrote: The boom of sciences drove a man into the tunnel of specialized disciplines [1]. With increasing specialization, many doctors come into a contact with the same patients only episodically. In addition, there are also time limits for individual patients. There is no time to apply knowledge across different levels and areas. The happiness of science is that there are no such limitations and our meeting is witnessing it. In contrast, research on nitric oxide (NO) demonstrates that a transdisciplinary approach is not only possible, but also necessary: in the case of NO it combines cardiovascular physiology, neuroscience, molecular biology, psychology and even noetics.

Nitric oxide (NO) in the brain is synthesized mainly by endothelial nitric oxide synthase (eNOS) and neuronal nitric oxide synthase (nNOS). It activates ion channels and regulates the expression of proteins important for axonal and dendritic growth, thereby supporting the formation of new neural connections crucial for learning and memory. NO mediates long-term potentiation (LTP) as a retrograde signal: formed in the postsynaptic membrane, it diffuses back to the presynaptic neuron, increasing neurotransmitter release and strengthening synaptic connections (the so-called Hebbian principle – neurons that fire together, wire together).

Through activation of guanylate cyclase, NO increases cGMP levels, which in turn activate protein kinase G (PKG), modulating ion channels and protein activity involved in synaptic plasticity and memory. Inhibition of nNOS after learning disrupts long-term memory consolidation, indicating its key role in transforming labile memory traces into more permanent ones [2,3].

The contribution of NO extends beyond synaptic plasticity. Changes in nNOS activity and glial reactivity in animal models of ADHD suggest that altered NO production may be associated with attention disorders. The coupling of attention and memory is strong, and NO acts as a neuromodulator affecting the release of various neurotransmitters, influencing mood, motivation and cognition. Older individuals with mild to moderate cognitive impairment show significantly lower levels of NO metabolites compared to healthy age-matched controls. This suggests that adequate NO availability supports attention, memory and cognitive functions, and may exert protective effects against neurodegenerative diseases [4,5].

As John Locke [6] formulated: Nothing exists in the mind which was not first in the senses. Sensualistic noetics, as the basis of modern philosophy, emphasizes experience as the source of knowledge and moral action. Research on NO extends this idea by uncovering biological mechanisms that enable sensory experience to be encoded, consolidated and integrated. NO provides the molecular basis for learning, memory and attention – the very tools through which philosophical reflection and knowledge are possible.

Thus, NO research illustrates the “mechanism of experience” at the level of synapses, neurons and vascular regulation, linking biology with cognition and higher intellectual functions. This clearly demonstrates that a transdisciplinary combination of medicine, neuroscience, psychology and philosophy can overcome the “tunnel vision” of specialization that Kundera criticized.
